# Real-world efficacy and safety of inetetamab-based therapy in HER2-positive metastatic breast cancer patients with prior exposure to trastuzumab

**DOI:** 10.3389/fonc.2025.1496371

**Published:** 2025-09-02

**Authors:** Yangyang Duan, Tao Sun, Zhanhong Chen, Quchang Ouyang, Kai Li, Min Yan, Zheng Lv, Zhaohui Li, Li Man, Xu Luo, Yuyang Dong, Mingxi Jing, Yan Wang, Xiangyu Guo, Junnan Xu, Xiaorui Li, Cui Jiang, Ying E, Lei Jiang, Hui Cao, Yufeng Jia, Jie Wu, Huan Li, Liang Zhang, Yujun Jiang, Zhichao Gao, Fangyuan Dong

**Affiliations:** ^1^ Department of Breast Medical Oncology, Liaoning Cancer Hospital and Institute, Shenyang, China; ^2^ Department of Breast Medical Oncology, Zhejiang Cancer Hospital, Hangzhou, China; ^3^ Breast Internal Medicine Department, Hunan Cancer Hospital, Changsha, China; ^4^ Department of Breast Medical Oncology, Shengjing Hospital of China Medical University, Shenyang, China; ^5^ Department of Breast Disease, The Affiliated Cancer Hospital of Zhengzhou University & Henan Cancer Hospital, Zhengzhou, China; ^6^ Department of Oncology, The First Hospital of Jilin University, Changchun, China; ^7^ Department of Breast Oncology, Anshan Cancer Hospital, Anshan, China

**Keywords:** breast cancer, inetetamab, HER2, monoclonal antibody, real-word data

## Abstract

**Background:**

Until now there has been no comprehensive data from extensive samples to evaluate the therapeutic potential of inetetamab in metastatic breast cancer (MBC) patients who had a history of trastuzumab treatment. Some previous studies had either small sample sizes from single-center, or partial enrolled patients without prior exposure to trastuzumab. This study aimed to provide a deep analysis of inetetamab-based therapy in this specific population.

**Methods:**

A multicenter retrospective study collected clinicopathological data from a total of 500 patients between Jul 2020 and Oct 2023. Progression-free survival (PFS) was estimated as the primary endpoint. Secondary endpoints included objective response rate (ORR), disease control rate (DCR) and adverse events (AEs). The association of risk factors with the effect of inetetamab treatment on PFS was evaluated using Cox proportional hazards regression analysis.

**Results:**

In the overall cohort, we observed a median PFS of 8.0 months, an ORR of 28.6% and a DCR of 89.2% (median treatment line: third line). Meanwhile, patients who received a combination treatment regime of inetetamab + tyrosine kinase inhibitors (TKIs) + chemotherapy achieved the most prolonged PFS of 9.0 months and the higher ORR of 29.3%. Ki67 index, distant lymph nodes metastasis and treatment strategy were independent predictors of PFS. The most common all-grade AEs were neutropenia (182/500, 36.4%) and leukopenia (157/500, 31.4%).

**Conclusions:**

Inetetamab was promising in HER2-positive MBC patients who had prior exposure to trastuzumab, offering a new option for patients with a prior failure of trastuzumab.

## 1 Introduction

According to the latest statistics from the World Health Organization (WHO), global new breast cancer cases surpassed 2.3 million in 2022, representing 11.7% of all cancer cases worldwide and claiming approximately 670,000 lives. It remains the most commonly diagnosed cancer among women globally (1). Data from the National Cancer Center of China indicate that in 2022, China recorded approximately 357,000 new breast cancer cases and about 75,000 deaths, posing a serious threat to women’s health in China (2). HER2 gene amplification or overexpression is found in 20%-30% of breast cancer cases (3). The overexpression of HER2 is closely correlated with the incidence, progression and outcome of breast cancer. Strong invasion, inadequate histological differentiation, and a high rate of metastasis and recurrence characterize HER2-positive breast cancer (4).

The recommended first-line therapy for HER2-positive metastatic breast cancer (MBC) involves trastuzumab in Chinese Society of Clinical Oncology (CSCO) breast cancer guideline. tyrosine kinase inhibitors (TKIs) or antibody-drug conjugate (ADC) have emerged as the second-line treatment strategy for HER2-positive MBC patients who have failed trastuzumab treatment in China (5). Despite the outstanding performance of ADCs, their high costs remain a significant burden for most Chinese MBC patients, particularly in economically underdeveloped regions, forcing many patients to forgo treatment due to financial constraints (6). Unfortunately, most Chinese patients who have failed both trastuzumab and TKIs could only have limited benefits from attempting trastuzumab-based regimens again (7).

Inetetamab is the first developed novel anti-HER2 monoclonal antibody in China, offering a more economical and effective therapy option (8, 9). A Phase III clinical trial known as HOPES study reported that inetetamab + vinorelbine could significantly prolong the progression-free survival (PFS) compared to chemotherapy alone (39.6 weeks vs. 14.0 weeks, p < 0.0001) (10). Subsequently, inetetamab has been approved by the China National Medical Products Administration (NMPA) for the treatment of HER2-positive MBC in June 2020. Meanwhile, the first-line subgroup analysis in this HOPES study showed that inetetamab exhibited a median PFS of 11.1 months for the first-line treatment in HER2-positive MBC patients, which was comparable to that of trastuzumab. Fortunately, the incidence of adverse events (AEs) did not increase significantly (11).

However, there had been a lack of comprehensive data from large sample sizes to evaluate inetetamab-based therapy in HER2-positive MBC patients who were previously treated with trastuzumab. The sample sizes in the previous studies were relatively small (12, 13). Not all enrolled patients had previously received trastuzumab treatment (12). And the study of Yu et al. was single-center (13). These factors resulted in several important limitations. Based on the current situation, this study with a larger-scale patients aimed to analyze the efficacy and safety of inetetamab-based therapy in HER2-positive MBC patients who had previously received trastuzumab treatment, exploring a more economical and effective therapeutic option.

## 2 Methods

### 2.1 Study design

A multi-center retrospective study, led by Liaoning Cancer Hospital & Institute in China from Jul 2020 to Oct 2023, was registered at https://www.ClinicalTrials.gov (NCT06305702). The informed consent was waived to be written as it solely utilized pre-existing data and records collected during the period of investigation.

### 2.2 Patients

The inclusion criteria were as follows: (I) an age of at least 18 years or older; (II) pathologically diagnosed HER2-positive MBC; (III) immunohistochemistry (IHC) 3+ or IHC 2+ with fluorescence *in situ* hybridization (FISH) positive; (IV) at least one measurable lesion as defined in the Response Criteria Evaluation in Solid Tumors version 1.1 (RECIST v1.1) (14); (V) prior trastuzumab therapy (≥1 cycle administered at any disease stage, either early or metastatic); (VI) receiving inetetamab-based therapy in the MBC stage; (VII) retaining traceable medical history records.

The exclusion criteria included: (I) pregnant or lactating women; (II) patients previously diagnosed with other malignant tumors; (III) patients with severe and uncontrolled infectious diseases, severe underlying medical conditions, or psychiatric illnesses; (IV) ineligible for this study by the researchers’ assessment.

### 2.3 Trial endpoints

The primary end point of the trial was PFS which was defined as the duration from randomization until first evidence of tumor progression or death from any cause. Secondary endpoints were objective response rate (ORR) which was defined as the proportion of patients that achieved a complete response (CR) or partial response (PR), and disease control rate (DCR) which was calculated as the percentage of patients whose therapeutic intervention had led to a CR, PR or stable disease (SD). RECIST v1.1 was used to assess the tumor treatment response in all the enrolled patients with measurable lesions (14). Other secondary endpoints encompassed AEs, which was estimated according to CTCAE version 5.0.

### 2.4 Procedures

Basic information, laboratory and imaging examination results, treatment history, tumor characteristics, metastatic sites, hormone receptor/HER2 status, etc. were retrospectively collected from the electronic medical record system in the involved hospitals. Treatment details, clinical efficacy assessments, safety evaluations, and other pertinent data from subsequent follow-up visits were also comprehensively gathered.

### 2.5 Statistical analysis

The Kaplan-Meier method was utilized to estimate the survival curves, and the Cox proportional hazards model was used to analyze hazard ratio (HR) with corresponding 95% confidence intervals (CIs). Relevant variables whose p values were < 0.2 in the univariate analysis were included into the multivariate regression model to determine risk factors for PFS. The relatively liberal threshold is recommended in the literature to avoid excluding potentially important predictors (12, 15). For the safety analysis, the numbers and percentages of patients with AEs of each grade were calculated. All statistical analyses were conducted using SAS version 9.4 and R software version 4.3.2.

## 3 Results

### 3.1 Patient characteristics

A total of 500 patients was enrolled in this study from Jul 2020 to Oct 2023. **Table 1** presented patient characteristics. The overall population had a median age of 54 years (range 26 to 85), with 394 patients (78.8%) being older than 45 years. Additionally, 461 patients (92.2%) had an Eastern Cooperative Oncology Group (ECOG) score of 0-1, and 337 patients (67.4%) were defined as IHC 3+. Lung was the most common observed metastatic site (233 patients, 46.6%), followed by local lymph nodes (217 patients, 43.4%), bone (214 patients, 42.8%), distant lymph nodes (206 patients, 41.2%), liver (182 patients, 36.4%) and brain (129 patients, 25.8%). In the previous treatment, all 500 enrolled patients had received trastuzumab (100%) and other anti-HER2 regimens including 152 patients (30.4%) with trastuzumab + pertuzumab, 113 patients (22.6%) with HER2-ADC, and 335 patients (67%) with TKIs.

**Table 1:** Patient characteristics (n = 500).

Variable	Inetetamab + Pertuzumab + Chemotherapy (n = 45, %)	Inetetamab + TKIs + Chemotherapy (n = 208, %)	Inetetamab + Chemotherapy (n = 247, %)	Total patients (n = 500, %)
Age (years)				
≤ 45	10 (22.2)	56 (26.9)	40 (16.2)	106 (21.2)
	35 (77.8)	152 (73.1)	207 (83.8)	394 (78.8)
ECOG score				
0-1	42 (93.3)	187 (89.9)	232 (93.9)	461 (92.2)
≥ 2	3 (6.7)	21 (10.1)	15 (6.1)	39 (7.8)
HER2 status				
3+	35 (77.8)	139 (66.8)	163 (66.0)	337 (67.4)
IHC 2+ and FISH-positive	10 (22.2)	69 (33.2)	84 (34.0)	163 (32.6)
Hormone receptor status				
Negative	27 (60.0)	93 (44.7)	119 (48.2)	239 (47.8)
Positive	18 (40.0)	115 (55.3)	128 (51.8)	261 (52.2)
Ki67				
< 14%	11 (24.4)	30 (14.4)	51 (20.6)	92 (18.4)
≥ 14%	34 (75.6)	178 (85.6)	196 (79.4)	408 (81.6)
Metastatic site				
Local lymph nodes	15 (33.3)	81 (38.9)	121 (49.0)	217 (43.4)
Distant lymph nodes	19 (42.2)	116 (55.8)	71 (28.7)	206 (41.2)
Brain	7 (15.6)	68 (32.7)	54 (21.9)	129 (25.8)
Lung	24 (53.3)	109 (52.4)	100 (40.5)	233 (46.6)
Bone	20 (44.4)	93 (44.7)	101 (40.9)	214 (42.8)
Liver	14 (31.1)	86 (41.3)	82 (33.2)	182 (36.4)
Radiation	17 (37.8)	114 (54.8)	107 (43.3)	238 (47.6)
Previous anti-HER2 treatment				
Trastuzumab	45 (100.0)	208 (100.0)	247 (100.0)	500 (100.0)
Trastuzumab + Pertuzumab	24 (54.5)	80 (40.0)	48 (19.4)	152 (30.4)
ADC	11 (25.0)	63 (31.5)	39 (15.8)	113 (22.6)
TKIs	31 (70.5)	150 (75.0)	154 (62.3)	335 (67)
Therapy lines				
1 line	1 (2.2)	39 (18.8)	25 (10.1)	65 (13.0)
2 lines	11 (24.4)	55 (26.4)	45 (18.2)	111 (22.2)
3 lines	14 (31.1)	39 (18.8)	91 (36.8)	144 (28.8)
4 lines	14 (31.1)	34 (16.3)	42 (17.0)	90 (18.0)
≥ 5 lines	5 (11.1)	41 (19.7)	44 (17.8)	90 (18.0)

ECOG, Eastern Cooperative Oncology Group; IHC, immunohistochemistry; FISH, fluorescence *in situ* hybridization; TKIs, tyrosine kinase inhibitors; ADC, antibody-drug conjugate.

### 3.2 Patient treatment

Most patients were given the chemotherapeutic agents plus anti-HER2 drugs (**Table 2**), including inetetamab + chemotherapy (247/500, 49.4%), inetetamab + TKIs + chemotherapy (208/500, 41.6%) and inetetamab + pertuzumab + chemotherapy (45/500, 9.0%). Meanwhile, most patients received vinorelbine (chemotherapy drug) (291/500, 58.2%), followed by paclitaxel (59/500, 11.8%), utidelone (38/500, 7.6%), eribulin (22/500, 4.4%), capecitabine (20/500, 4.0%), and others (70/500, 14.0%).

**Table 2:** Treatment administration.

Chemotherapy	Inetetamab + Pertuzumab + Chemotherapy (n = 45, %)	Inetetamab + TKIs + Chemotherapy (n = 208, %)	Inetetamab + Chemotherapy (n = 247, %)	Total Patients (n = 500, %)
Vinorelbine	32 (71.1)	87 (41.8)	172 (69.6)	291 (58.2)
Paclitaxel	9 (20.0)	29 (13.9)	21 (8.5)	59 (11.8)
Eribulin	0 (0.0)	14 (6.7)	8 (3.2)	22 (4.4)
Capecitabine	0 (0.0)	16 (7.7)	4 (1.6)	20 (4.0)
Utidelone	1 (2.2)	37 (17.8)	0 (0.0)	38 (7.6)
Others	3 (6.7)	25 (12.0)	42 (17.0)	70 (14.0)

TKIs, tyrosine kinase inhibitors.

### 3.3 Efficacy in overall population

**Figure 1:**
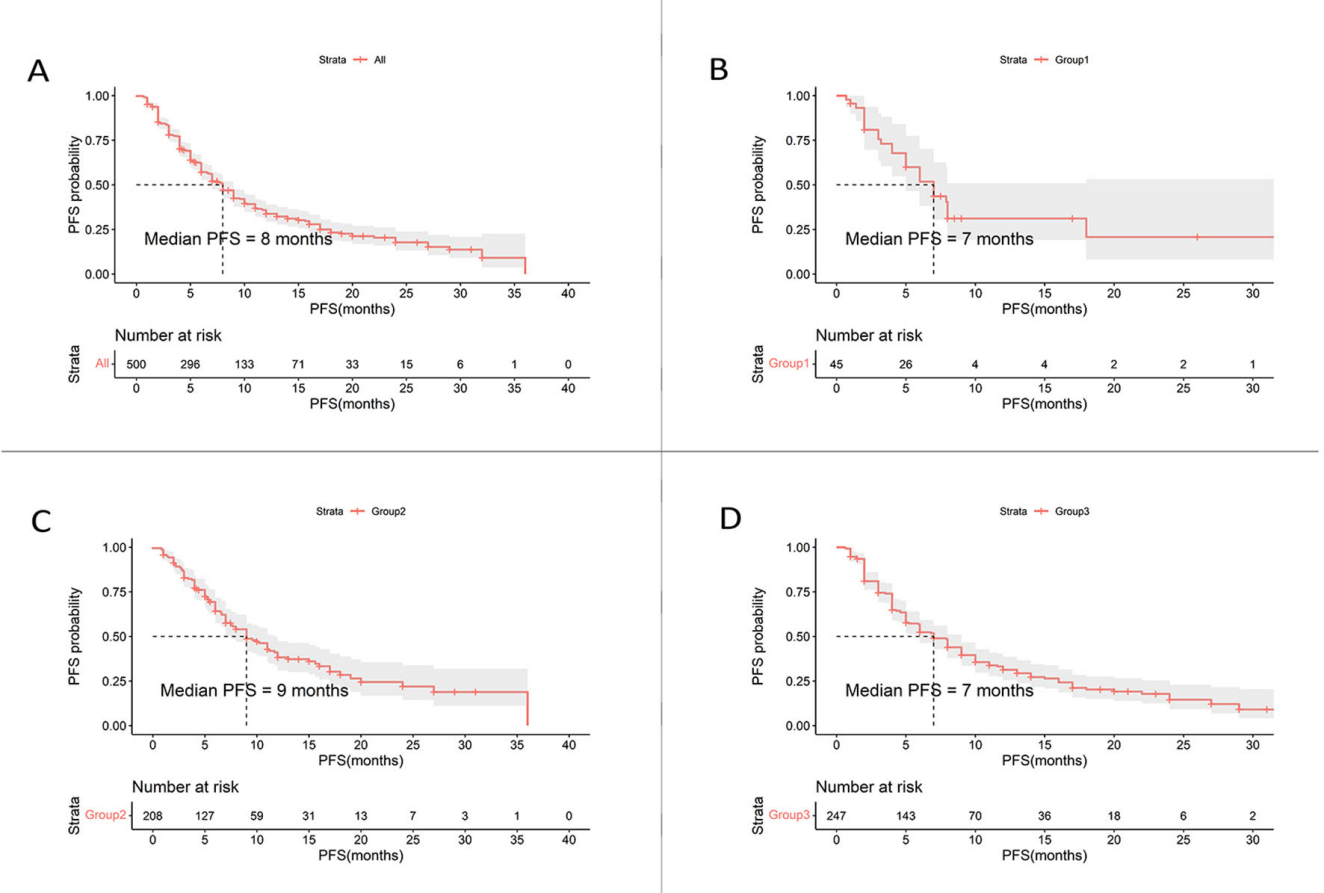
Kaplan-Meier curves of PFS for HER2-positive MBC patients. **(A)** Kaplan-Meier estimates for the entire cohort (n = 500). **(B)** Kaplan-Meier estimates for patients treated with inetetamab + pertuzumab + chemotherapy (n = 45). **(C)** Kaplan-Meier estimates for patients treated with inetetamab + TKIs + chemotherapy (n = 208). **(D)** Kaplan-Meier estimates for patients treated with inetetamab + chemotherapy (n = 247). PFS, progression-free survival; TKIs, tyrosine kinase inhibitors.

The median PFS was 8.0 months in the overall cohort (**Table 3**, **Figure 1A**). Of the 500 included patients, there were 2 patients (0.4%) with CR, 141 patients (28.2%) with PR, 303 patients (60.6%) with SD, and 52 patients (10.4%) with progressive disease (PD). Overall, the ORR and DCR were calculated to be 28.6% and 89.2%, respectively.

**Table 3:** Evaluation of efficacy.

Variable	Inetetamab + Pertuzumab + Chemotherapy (n = 45, %)	Inetetamab + TKIs + Chemotherapy (n = 208, %)	Inetetamab + Chemotherapy (n = 247, %)	Total Patients (n = 500, %)
Median of lines	3	3	3	3
Median PFS (months)	7.0	9.0	7.0	8.0
Events No. (%)	28 (62.2)	111 (53.4)	171 (69.2)	310 (62.0)
Best overall response no. (%)				
CR	0 (0.0)	1 (0.5)	1 (0.4)	2 (0.4)
PR	11 (24.4)	60 (28.8)	70 (28.3)	141 (28.2)
SD	29 (64.4)	132 (63.5)	142 (57.5)	303 (60.6)
PD	5 (11.1)	13 (6.2)	34 (13.8)	52 (10.4)
NE	0 (0.0)	2 (1.0)	0 (0.0)	2 (0.4)
ORR No. (%)	11 (24.4)	61 (29.3)	71 (28.7)	143 (28.6)
DCR No. (%)	40 (88.9)	193 (92.8)	213 (86.2)	446 (89.2)

PFS, progression-free survival; ORR, objective response rate; DCR, disease control rate; FISH, fluorescence *in situ* hybridization; TKIs, tyrosine kinase inhibitors; CR, complete response; PR, partial response; SD, stable disease; PD, progressive disease; NE, not evaluable.

Patients who received inetetamab + TKIs + chemotherapy treatment achieved the most prolonged PFS of 9.0 months, which surpassed those of other regimens (**Figures 1B–D**) including inetetamab + pertuzumab + chemotherapy (7.0 months) as well as inetetamab + chemotherapy (7.0 months). The ORRs for the various treatment regimens were listed as follows: inetetamab + TKIs + chemotherapy (29.3%), inetetamab + chemotherapy (28.7%), and inetetamab + pertuzumab + chemotherapy (24.4%). Additionally, the combination treatment of inetetamab + TKIs + chemotherapy demonstrated an outstanding DCR of 92.8%, followed by inetetamab + pertuzumab + chemotherapy (88.9%) as well as inetetamab + chemotherapy (86.2%).

### 3.4 Subgroup analysis of PFS based on inetetamab + TKIs + chemotherapy

As mentioned above, patients who received inetetamab + TKIs + chemotherapy treatment achieved a longer PFS of 9.0 months. To explore determinants of this clinical benefit, we conducted both univariate and multivariate analyses across common baseline variables (**Table 4**).

**Table 4:** Subgroup analysis of PFS based on inetetamab + TKIs + chemotherapy treatment.

Variable	n	Median PFS (months)	Log-rank *p* value	Univariate		Multivariate	
				HR (95% CI)	*p* value	HR (95% CI)	*p* value
Age (years)							
≤ 45	56	8.0	0.385	0.840 (0.564-1.252)	0.393		
> 45	152	10.0					
ECOG score							
0-1	187	9.0	0.343	1.210 (0.874-1.675)	0.250		
2	21	9.0					
HER2 status							
IHC 3+	139	9.56	0.368	0.837 (0.565-1.241)	0.376		
IHC 2+ and FISH-positive	69	7.0					
Hormone receptor status							
Negative	93	8.0	0.934	1.016 (0.695-1.485)	0.935		
Positive	115	9.56					
Ki67							
< 14%	30	11.0	0.147	1.470 (0.863-2.504)	0.156	1.658 (0.883-3.113)	0.115
≥ 14%	178	9.0					
Metastatic site							
Local lymph nodes (yes vs. no)	81 vs. 127	11.7 vs. 9.0	0.497	0.876 (0.593-1.293)	0.504		
Distant lymph nodes (yes vs. no)	116 vs. 92	9.0 vs. 10.0	0.201	1.278 (0.872-1.875)	0.209		
Brain (yes vs. no)	68 vs. 140	7.0 vs. 11.0	0.114	1.353 (0.923-1.982)	0.121	1.067 (0.682-1.670)	0.776
Lung (yes vs. no)	109 vs. 99	11.0 vs. 8.0	0.376	0.847 (0.582-1.232)	0.384		
Bone (yes vs. no)	93 vs. 115	7.5 vs. 11.2	0.017	1.563 (1.073-2.275)	0.020	1.467 (0.957-2.247)	0.079
Liver (yes vs. no)	86 vs. 122	6.6 vs. 11.2	0.000	2.033 (1.396-2.963)	0.000	1.630 (1.057-2.515)	0.027
Radiation (yes vs. no)	114 vs. 94	7.0 vs. 12.0	0.012	1.625 (1.101-2.396)	0.014	1.660 (1.056-2.611)	0.028
Previous anti-HER2 treatment							
Trastuzumab +Pertuzumab (yes vs. no)	80 vs. 120	9.0 vs. 11.0	0.058	1.444 (0.980-2.126)	0.063	1.578 (1.044-2.385)	0.031
ADC (yes vs. no)	63 vs 137	8.0 vs. 11.0	0.024	1.556 (1.051-2.305)	0.027	1.239 (0.775-1.982)	0.371
TKI (yes vs. no)	150 vs 50	9.0 vs. 12.0	0.111	1.471 (0.905-2.393)	0.120	1.513 (0.880-2.601)	0.134
Therapy line			0.019				
1 line	39	11.0		0.797 (0.408-1.557)	0.507	0.780 (0.352-1.731)	0.541
2 lines	55	9.0		0.970 (0.547-1.722)	0.918	1.075 (0.562-2.055)	0.828
3 lines	39	10.2		0.936 (0.510-1.719)	0.831	0.819 (0.415-1.618)	0.566
4 lines	34	6.0		1.934 (1.081-3.462)	0.026	2.133 (1.122-4.054)	0.021
≥ 5 lines	41	10.0		Ref	Ref		
Combined chemotherapy drugs							
Vinorelbine	87	10.2	0.674	0.715 (0.409-1.250)	0.240	0.619 (0.337-1.136)	0.122
Paclitaxel	29	11.0		0.633 (0.319-1.256)	0.191	0.465 (0.226-0.957)	0.038
Eribulin	14	8.0		0.638 (0.234-1.734)	0.378	0.708 (0.254-1.973)	0.509
Capecitabine	16	12.0		0.569 (0.245-1.323)	0.190	0.552 (0.222-1.372)	0.201
Utidelone	37	9.0		0.608 (0.309-1.195)	0.149	0.661 (0.297-1.472)	0.311
Others	25	6.0		Ref	Ref		

ECOG, Eastern Cooperative Oncology Group; PFS, progression-free survival; TKIs, tyrosine kinase inhibitors; ADC, antibody-drug conjugate; IHC, immunohistochemistry; FISH, fluorescence *in situ* hybridization; HR, Hazard ratio; CI, Confidence interval; Ref, reference.

**Figure 2:**
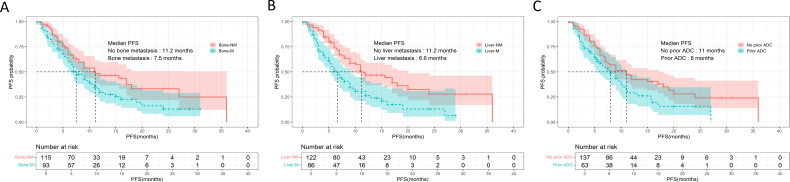
Kaplan-Meier curves of PFS for patients treated with inetetamab + TKIs + chemotherapy. **(A)** PFS analysis of patients with (n = 93) and without (n = 115) bone metastasis. **(B)** PFS analysis of patients with (n = 86) and without (n = 122) liver metastasis. **(C)** PFS analysis of patients with (n = 63) and without (n = 137) prior ADC treatment. PFS, progression-free survival; TKIs, tyrosine kinase inhibitors.

Univariate screening identified bone metastasis (7.5 vs. 11.2 months, *p* = 0.017), liver metastasis (6.6 vs. 11.2 months, *p* < 0.001), prior ADC exposure (8.0 vs. 11.0 months, *p* = 0.024), radiotherapy history (7.0 vs. 12.0 months, *p* = 0.012) and therapy line (*p* = 0.019) as factors significantly associated with PFS (**Figure 2**). Variables with *p* < 0.20 were entered into a multivariate Cox model, whitch additionally included Ki-67 index, brain metastasis, prior trastuzumab + pertuzumab, prior TKI use, and the specific cytotoxic partner.

After adjustment, liver metastasis [HR 1.630, 95% CI 1.057-2.515; *p* = 0.027], history of radiotherapy [HR 1.660, 95% CI 1.056-2.611; *p* = 0.028], fourth-line vs. ≥ fifth-line therapy [HR 2.133, 95% CI 1.122-4.054; *p* = 0.021], and prior trastuzumab + pertuzumab treatment [HR 1.578, 95% CI 1.044 -2.385; *p* = 0.031] remained independently predictive of shorter PFS. Conversely, paclitaxel as the chemotherapy backbone was independently associated with prolonged PFS [HR 0.465, 95% CI 0.226-0.957, *p* = 0.038].

### 3.5 Cox regression analysis of PFS

The univariate analysis (**Table 5**) indicated that Ki67 index, distant lymph nodes metastasis, previous TKIs treatment and treatment strategy were significantly associated with the PFS (all *p* values < 0.05). Meanwhile, relevant variables that exhibited *p*-values less than 0.2 in the univariate analysis were exported into the multivariate regression model, including age, HER2 expression, Ki67 index, distant lymph nodes metastasis, brain metastasis, bone metastasis, liver metastasis, a history of radiation therapy, previous anti-HER2 treatment (TKIs or ADC), and treatment strategy (inetetamab + TKIs + chemotherapy). The Cox multivariate regression results revealed that the following factors were independently predictive of PFS: Ki67 index [HR 1.361, 95% CI 1.004-1.846; *p* = 0.047], distant lymph nodes metastasis [HR 1.437, 95% CI 1.126-1.835; *p* = 0.004], and treatment strategy (inetetamab + TKIs + chemotherapy) [HR 0.607, 95% CI 0.469-0.787; *p* < 0.000].

**Table 5:** Univariate and multivariate analyses of factors predicting PFS.

Variable	Median PFS (months)	Log-rank *p* value	Univariate		Multivariate	
			HR (95% CI)	*p* value	HR (95% CI)	*p* value
Age (years)						
≤ 45	6	0.164	0.836 (0.644-1.084)	0.177	0.997 (0.985-1.009)	0.592
> 45	8		Ref	Ref		
ECOG score						
0-1	8	0.374	1.105 (0.904-1.352)	0.329		
2	6		Ref	Ref		
HER2 status						
IHC 3+	9	0.135	0.840 (0.664-1.063)	0.147	0.811(0.636-1.035)	0.093
IHC 2+ and FISH-positive	7		Ref	Ref		
Hormone receptor status						
Negative	7	0.663	0.953(0.761-1.192)	0.672		
Positive	8		Ref	Ref		
Ki67 index						
< 14%	9	0.018	1.407(1.051-1.884)	0.022	1.361 (1.004-1.846)	0.047
≥ 14%	7		Ref	Ref		
Metastatic site						
Local lymph nodes (yes vs. no)	8.0 vs. 7.7	0.363	0.903 (0.720-1.132)	0.378		
Distant lymph nodes (yes vs. no)	7.0 vs. 9.0	0.034	1.266 (1.010-1.586)	0.041	1.437 (1.126-1.835)	0.004
Brain (yes vs. no)	6.0 vs. 8.0	0.089	1.232 (0.961-1.579)	0.099	1.174 (0.899-1.533)	0.239
Lung (yes vs. no)	8.0 vs. 7.9	0.481	0.925 (0.739-1.157)	0.500		
Bone (yes vs. no)	7.0 vs. 9.0	0.135	1.180 (0.943-1.477)	0.148	1.052 (0.824-1.343)	0.684
Liver (yes vs. no)	7.0 vs. 8.0	0.078	1.222 (0.971-1.537)	0.087	1.141 (0.893-1.458)	0.292
Radiation (yes vs. no)	7.0 vs. 9.0	0.119	1.189 (0.951-1.488)	0.129	1.075 (0.848-1.362)	0.550
Previous anti-HER2 treatment						
Trastuzumab + Pertuzumab (yes vs. no)	8.0 vs. 8.0	0.231	1.155 (0.906-1.473)	0.245		
ADC (yes vs. no)	8.0 vs. 8.0	0.062	1.271 (0.980-1.650)	0.071	1.166 (0.888-1.531)	0.270
TKIs (yes vs. no)	7.9 vs. 8.0	0.043	1.286 (1.000-1.655)	0.050	1.293 (0.994-1.681)	0.056
Therapy line						
1 line	11.0	0.141	0.734 (0.472-1.140)	0.168		
2 lines	9.0		0.756 (0.525-1.090)	0.135		
3 lines	7.0		1.018 (0.734-1.412)	0.916		
4 lines	7.7		1.062 (0.741-1.523)	0.743		
≥ 5 lines	6		Ref	Ref		
Treatment strategy		0.032				
Inetetamab + Pertuzumab + Chemotherapy	7		1.081 (0.724-1.615)	0.704	1.027 (0.674-1.565)	0.901
Inetetamab + TKIs + Chemotherapy	9		0.751 (0.591-0.954)	0.019	0.607 (0.469-0.787)	0.000
Inetetamab + Chemotherapy	7		Ref	Ref		
Combined chemotherapy drugs		0.457				
Vinorelbine	7.9		1.146 (0.829-1.585)	0.409		
Paclitaxel	7.0		1.124 (0.738-1.713)	0.586		
Eribulin	8.0		0.825 (0.389-1.751)	0.616		
Capecitabine	12.0		0.876 (0.463-1.656)	0.683		
Utidelone	9.0		0.749 (0.429-1.309)	0.310		
Others	7.0		Ref	Ref		

ECOG, Eastern Cooperative Oncology Group; PFS, progression-free survival; TKIs, tyrosine kinase inhibitors; ADC, antibody-drug conjugate; IHC, immunohistochemistry; FISH, fluorescence *in situ* hybridization; HR, Hazard ratio; CI, Confidence interval; Ref, reference.

### 3.6 Safety

The most common all-grade AEs (**Table 6**) in all the enrolled patients were neutropenia (182/500, 36.4%) and leukopenia (157/500, 31.4%), followed by increased transaminase (105/500, 21.0%), anemia (71/500, 14.2%), and diarrhea (66/500, 13.2%). Among them, the most common grades 3/4 AEs included neutropenia (26/500, 5.2%), leukopenia (22/500, 4.4%), thrombocytopenia (9/500, 1.8%), anemia (6/500, 1.2%), and GGT increased (5/500, 1.0%). No treatment-related deaths occurred. No specific cardiotoxicity was observed with inetetamab, including decreased left ventricular ejection fraction (LVEF) and congestive heart failure (CHF).

**Table 6:** Adverse events of all grades.

AEs, n (%)	Grade 1	Grade 2	Grade 3	Grade 4	All grade	Grade 3/4
Neutropenia	95 (19.0)	61 (12.2)	13 (2.6)	13 (2.6)	182 (36.4)	26 (5.2)
Leukopenia	96 (19.2)	39 (7.8)	9 (1.8)	13 (2.6)	157 (31.4)	22 (4.4)
Anemia	39 (7.8)	26 (5.2)	4 (0.8)	2 (0.4)	71 (14.2)	6 (1.2)
Thrombocytopenia	34 (6.8)	4 (0.8)	8 (1.6)	1 (0.2)	47 (9.4)	9 (1.8)
Fever	0 (0)	3 (0.6)	0 (0)	0 (0)	3 (0.6)	0 (0)
Fatigue	6 (1.2)	2 (0.4)	1 (0.2)	0 (0)	9 (1.8)	1 (0.2)
Pain	0 (0)	1 (0.2)	0 (0)	0 (0)	1 (0.2)	0 (0)
Increased transaminase	92 (18.4)	13 (2.6)	0 (0)	0 (0)	105 (21.0)	0 (0)
Increased blood bilirubin	16 (3.2)	4 (0.8)	0 (0)	0 (0)	20 (4.0)	0 (0)
ALP increased	13 (2.6)	2 (0.4)	3 (0.6)	0 (0)	18 (3.6)	3 (0.6)
GGT increased	22 (4.4)	4 (0.8)	5 (1.0)	0 (0)	31 (6.2)	5 (1.0)
Nausea	21 (4.2)	6 (1.2)	0 (0)	0 (0)	27 (5.4)	0 (0)
Vomiting	13 (2.6)	4 (0.8)	0 (0)	0 (0)	17 (3.4)	0 (0)
Diarrhea	56 (11.2)	8 (1.6)	2 (0.4)	0 (0)	66 (13.2)	2 (0.4)
Constipation	12 (2.4)	0 (0)	0 (0)	0 (0)	12 (2.4)	0 (0)
Abdominalgia	13 (2.6)	0 (0)	0 (0)	0 (0)	13 (2.6)	0 (0)
Abdominal distension	5 (1.0)	1 (0.2)	0 (0)	0 (0)	6 (1.2)	0 (0)
Oral ulcer	0 (0)	1 (0.2)	0 (0)	0 (0)	1 (0.2)	0 (0)
Dizziness	9 (1.8)	1 (0.2)	0 (0)	0 (0)	10 (2.0)	0 (0)
ECG abnormality	0 (0)	1 (0.2)	0 (0)	0 (0)	1 (0.2)	0 (0)
Cardiopalmus	1 (0.2)	2 (0.4)	0 (0)	0 (0)	3 (0.6)	0 (0)
Cough	16 (3.2)	0 (0)	0 (0)	0 (0)	16 (3.2)	0 (0)
Infection	0 (0)	2 (0.4)	0 (0)	0 (0)	2 (0.4)	0 (0)
Hand-foot syndrome	18 (3.6)	3 (0.6)	1 (0.2)	0 (0)	22 (4.4)	1 (0.2)
Peripheral neuropathy	52 (10.4)	8 (1.6)	0 (0)	0 (0)	60 (12.0)	0 (0)
Thrombosis	0 (0)	1 (0.2)	0 (0)	0 (0)	1 (0.2)	0 (0)
Hyperglycemia	0 (0)	0 (0)	1 (0.2)	0 (0)	1 (0.2)	1 (0.2)
Rash	1 (0.2)	0 (0)	0 (0)	0 (0)	1 (0.2)	0 (0)
Increased creatinine	1 (0.2)	1 (0.2)	0 (0)	0 (0)	2 (0.4)	0 (0)
Bleeding	0 (0)	1 (0.2)	0 (0)	0 (0)	1 (0.2)	0 (0)

AEs, adverse events; ALP, alkaline phosphatase; GGT, γ-glutamyl transpeptidase; ECG, electrocardiogram.

## 4 Discussion

In recent years, the proportion of patients who received trastuzumab in the neoadjuvant stage has reached up to 80% and will continue to rise in the future (5). This trend suggests that HER2-positive MBC patients treated with trastuzumab will also gradually increase. Currently, this was the first multi-center real-world study to directly investigate inetetamab-based therapy in HER2-positive MBC patients, all of whom had prior exposure to trastuzumab. Although inetetamab-based treatment had been evaluated in the previous studies, their sample sizes were relatively small (n ≤ 141) (12, 13). And not all enrolled patients had previously received trastuzumab (12). The study led by Yu et al. was single-center (13). These factors limited the previous studies to fully understand the efficacy and safety of inetetamab-based therapy in patients who had received trastuzumab. Therefore, we carried out this multi-center retrospective real-world study with larger-scale patients (n = 500). Specifically, all the enrolled patients had prior exposure to trastuzumab therapy.

Our study demonstrated that inetetamab-based treatment exhibited significant clinical efficacy for HER2-positive MBC patients, especially in prolonging PFS that was comparable or superior to current mainstream anti-HER2 regimens. All enrolled patients had prior trastuzumab treatment, with the majority receiving TKIs and/or ADCs. Despite the heavily pretreated patients (median treatment line: third line), the inetetamab combination achieved a median PFS of 8.0 months. We conducted an indirect comparative analysis with key HER2-targeted therapies. The NCCN-recommended third-line standard regimen of trastuzumab + tucatinib + capecitabine demonstrated a median PFS of 7.8 months in the HER2CLIMB trial (16, 17), whereas inetetamab-based therapy reached 8.0 months, suggesting a potential advantage in patients with extensive prior trastuzumab exposure.

Importantly, benefit was consistent across multiple treatment lines (18). As first-line treatment, the regimen achieved a median PFS of 11.0 months. Although lower than the 18.7 months with pertuzumab + trastuzumab + docetaxel (CLEOPATRA trial), this result is remarkable given all patients with prior trastuzumab exposure. In second-line therapy, median PFS was 9.0 months, surpassing the 6.9 months reported for T-DM1 (EMILIA trial). Third-line use yielded 7.0 months, comparable to the tucatinib regimen. Fourth-line administration achieved 7.7 months, exceeding the 6.9 months in the control group (trastuzumab/lapatinib + capecitabine, DESTINY-Breast02 trial). Even in fifth-line or beyond, median PFS was maintained at 6.0 months. These data demonstrate sustained antitumor activity and position inetetamab as a viable option for HER2-positive MBC after trastuzumab exposure.

Multivariate analysis of the overall study population confirmed that the treatment strategy of inetetamab + TKIs + chemotherapy was an independent protective factor for PFS (HR 0.607, 95% CI 0.469-0.787; *p* < 0.001). It revealed that the combination regimen achieved a median PFS of 9 months, an ORR of 29.3%, and a DCR of 92.8%, aligning with the 8.2 months reported by Liu et al. (12). Further multivariate Cox analysis within the inetetamab + TKIs + chemotherapy subgroup indicated that patients with absence of liver metastasis, no prior trastuzumab + pertuzumab therapy, no radiotherapy history, and those receiving paclitaxel-based chemotherapy derived significantly greater PFS benefits. The favorable outcome observed in the ≥ 5-line subgroup potentially reflects the regimen’s particular efficacy in highly selected late-line patients.

Although the median PFS was numerically longer in the overall population receiving capecitabine (12 months), univariate analysis indicated that no single chemotherapeutic agent had a statistically significant impact on PFS (all *p* > 0.20). Sensitivity analyses further suggested that heterogeneity in chemotherapy regimens could still confound inter-group comparisons (**Supplementary Material**, **Supplementary Tables S1**, **S2**). Therefore, multivariate models require simultaneous adjustment for key prognostic variables to accurately assess treatment differences. In the multivariate analysis of the inetetamab + TKIs + chemotherapy subgroup, capecitabine did not demonstrate an independent benefit (*p* = 0.201), whereas paclitaxel was independently associated with significantly prolonged PFS (*p* = 0.038).

Mechanistically, inetetamab acts extracellularly by blocking HER2 ligand binding, mediating antibody-dependent cellular cytotoxicity (ADCC), and promoting receptor internalization and degradation. TKI acts intracellularly by irreversibly inhibiting HER2/EGFR kinase activity, thereby blocking downstream PI3K/AKT and MAPK signaling pathways. Paclitaxel stabilizes microtubules, arrests mitosis, and induces mitochondrial apoptosis. Furthermore, its ability to upregulate HER2 expression enhances the targeted binding efficiency of inetetamab, creating a therapeutic positive feedback loop. Thie synergistic action is expected to further enhance anti-tumor efficacy (19, 20). The PHILA phase III study demonstrated that adding pyrotinib to trastuzumab + docetaxel significantly extended the median PFS from 10 to 24 months (21). Therefore, the inetetamab + TKIs + paclitaxel regimen warrants further validation in prospective studies.

Additionally, analysis of the overall population identified Ki-67 index and distant lymph node metastasis as independent predictors of PFS. Elevated Ki67 index, an indicative of rapid tumor proliferation and high malignancy, contributed to increased treatment challenges and the risk of drug resistance, thereby exhibiting a negative correlation with PFS. Moreover, distant lymph node metastasis means the dissemination of breast cancer, necessitating complex treatment strategies and often predicting a poor prognosis. Thus, monitoring Ki67 index and accurately assessing the status of lymph node metastasis are of paramount importance for tailoring treatment plans and better patient outcomes. Brain, lung and liver were the most common metastatic sites in HER2-positive breast cancer patients (22). But in contrast to several previous studies (13, 23–26), our study found that these metastatic sites were not significant predictors of PFS. This discrepancy could be attributed to differences in patient characteristics, study design or sample size. Only in the subgroup analysis was liver metastasis confirmed as an independent risk factor for shorter PFS (*p* = 0.027), while bone metastasis and prior ADC exposure lost significance in the multivariable model (*p* > 0.05). This indicates that the univariate PFS differences observed for the latter two variables are driven mainly by confounding effects of tumor burden and treatment history rather than by independent biological interactions.

The study identified a manageable spectrum of treatment-emergent adverse events, most of which were not attributed to inetetamab. Importantly, no cardiotoxicity was observed including LVEF decline or CHF, yielding a cardiac safety advantage over trastuzumab + anthracyclines (11.3% events: 8.7% LVEF decline, 2.35% CHF and over T-DXd in DESTINY-Breast03 (2.3% asymptomatic LVEF decline) (18). Classic HER2-targeted toxicities were also markedly reduced. Diarrhea and rash were predominantly low-grade; hepatic enzyme elevations were mild; and no cases of interstitial lung disease were observed, in contrast to the approximately 15.4% incidence reported with T-DXd (18). Ocular toxicities and severe hypersensitivity reactions were not observed, and hematologic events were limited to grade 1–2 neutropenia and leukopenia, without necessitating treatment discontinuation. Overall, inetetamab demonstrated a favorable safety profile in patients with HER2-positive MBC, supporting its potential as a well-tolerated and effective therapeutic option in this population.

Economic considerations further support the adoption of inetetamab-based regimens. Trastuzumab (Herceptin^®^) has undergone multiple price reductions since its launch in China over 20 years ago. Following its inclusion in the 2017 National Reimbursement Drug List (NRDL), the price for 440 mg/vial formulation plummeted by 78% (from RMB 24,500 to RMB 5,500). Nonetheless, it remains a heavy financial burden for most Chinese patients. Similarly, T-DXd (Enhertu^®^) is currently priced at RMB 3,480 for 100 mg/vial formulation. In contrast, the domestic anti-HER2 monoclonal antibody inetetamab was approved in June 2020 and entered the NRDL in 2021, offering 150 mg/vial (RMB 1,368) and 50 mg/vial (RMB 590). These flexible strengths enable precise weight-based dosing, eliminate wastage, and substantially ease both financial and clinical burdens.

There are some limitations in our study. As a multicenter retrospective analysis, it is susceptible to potential information bias and incomplete medical records, which restricted the number of covariates incorporated into the Cox model. Additionally, due to feasibility and time constraints, OS was not systematically collected during the design phase, with PFS serving as the sole primary efficacy endpoint. To minimize bias, we implemented stringent eligibility criteria and harmonized data-capture protocols across all participating centers. Subsequent manual verification and systematic data-cleaning eliminated erroneous or inconsistent records, ensuring enhanced data accuracy and consistency.

In conclusion, this large-scale, multi-center real-world study of 500 HER2-positive MBC patients demonstrated that inetetamab-based regimens deliver consistent PFS benefits with a favorable safety profile. Prospective randomized trials comparing inetetamab + TKIs + paclitaxel against alternative regimens, enhanced by biomarker-guided patient stratification, are now imperative for advancing personalized care paradigms.

## Data Availability

The original contributions presented in the study are included in the article/**Supplementary Material**, further inquiries can be directed to the corresponding author/s.
