# Supplementary material for: Real-world efficacy and safety of inetetamab-based therapy in HER2-positive metastatic breast cancer patients with prior exposure to trastuzumab
Source: Front Oncol. 2025 Sep 2;15:1496371. doi: 10.3389/fonc.2025.1496371 (PMC12436387; doi:10.3389/fonc.2025.1496371)
Supplement: Supplementary file 1 [file Table1.docx]

**Table S1. Sample size and median PFS by chemotherapeutic agent stratification**

| **Stratification** | **Inetetamab + Pertuzumab + Chemotherapy**  **(n = 45)** | **Inetetamab + TKIs + Chemotherapy**  **(n = 208)** | **Inetetamab + Chemotherapy**  **(n = 247)** |
| --- | --- | --- | --- |
| **Vinorelbine** |  |  |  |
| N | 32 | 87 | 172 |
| Median PFS (months) | 7 | 10.2 | 7 |
| **Paclitaxel (albumin-bound paclitaxel or liposomal paclitaxel)** | |  |  |
| N | 9 | 29 | 21 |
| Median PFS (months) | 6 | 11 | 6 |
| **Eribulin** |  |  |  |
| N | 0 | 14 | 8 |
| Median PFS (months) | -- | 8 | NA |
| **Capecitabine** |  |  |  |
| N | 0 | 16 | 4 |
| Median PFS (months) | -- | 12 | 8 |
| **Utidelone** |  |  |  |
| N | 1 | 37 | 0 |
| Median PFS (months) | NA | 9 | -- |
| **Others** |  |  |  |
| N | 3 | 25 | 42 |
| Median PFS (months) | 8 | 6 | 9 |

NA, not applicable; PFS, progression-free survival; TKIs, tyrosine kinase inhibitors.

**Table S2. Sensitivity analysis of PFS by Log-rank test (adjusted for chemotherapy agent)**

|  | **n** | **Median PFS**  **(months)** | **Unadjusted Log-rank**  ***p* value** | **Adjusted *p* value** |
| --- | --- | --- | --- | --- |
| **Treatment strategy** |  |  | **0.032*** | **0.173** |
| Inetetamab + Pertuzumab + Chemotherapy | 45 | 7 | Grp1 vs. Grp2#  0.031 | Grp1 vs. Grp2#  0.185 |
| Inetetamab + TKIs + Chemotherapy | 208 | 9 | Grp2 vs. Grp3#  0.045 | Grp2 vs. Grp3#  0.195 |
| Inetetamab + Chemotherapy | 247 | 7 |  |  |

PFS, progression-free survival; TKIs, tyrosine kinase inhibitors.

^*^ Log-rank overall comparison P value; ^#^ Pairwise comparisons adjusted by Tukey-Kramer correction.
